# Supplementary material for: Different patterns of pneumothorax in patients with soft tissue tumors treated with pazopanib: A case series analysis
Source: PLoS One. 2021 Jul 16;16(7):e0254866. doi: 10.1371/journal.pone.0254866 (PMC8284672; doi:10.1371/journal.pone.0254866)
Supplement: S1 Table — (DOCX) [file pone.0254866.s001.docx]

S1 Table. Summary of patient characteristics and treatment courses.

| **Number** | **Age**  **(year)** | **Histology** | **Extrapulmonary metastases** | **Previous lung treatment before PZP administration** | **PZP dose (mg)** | **Number of lung metastases**  **(subpleural / central type)** | **Best response** | **Cavitation (time to cavitation)** | **Types of PTX** | **Time to PTX (after PZP intiation) and severity** | **Treatment of PTX** | **Re-administration of PZP** | **Total treatment periods of PZP (months)** |
| --- | --- | --- | --- | --- | --- | --- | --- | --- | --- | --- | --- | --- | --- |
| 1 | 66 | UPS | Soft tissue | VATS | 600 | 1 (0/1) | PR | +, 1m | Central type | 20m (complete) | Drainage → VATS | No | 20 |
| 2^*1^ | 73 | UPS | Pericardium | RFA | 800 | 7 (1/6) | PR | +, preexist | Central type  Central type | 3m (complete)  9m (complete) | Drainage → pleurodesis  Unknown (treated in different hospital) | Yes | 8 |
| 3 | 57 | UPS | Brain | VATS | 800 | 27 (12/15) | PR | +, 2w | Subpleural type  Subpleural type  Subpleural type  Subpleural type  Subpleural type | 3w (moderate)  2m (small)  3m (moderate)  6m (moderate)  7m (complete) | Drainage → pleurodesis  Observation  Drainage → pleurodesis  Drainage → pleurodesis  Drainage | Yes | 6 |
| 4^*2^ | 15 | exOS | Bone | No | 800 | 48 (30/18) | PR | +, 2w | Subpleural type  Subpleural type  Subpleural type  Subpleural type  Subpleural type  Subpleural type | 2w (moderate)  1m (small)  3m (moderate)  4m (moderate)  5m (moderate)  6m (moderate) | Drainage  Observation  Drainage  Drainage → pleurodesis  Drainage  Drainage | Yes | 4 |
| 5 | 76 | LMS | Soft tissue | RFA | 800 | 8 (4/4) | PR | +, 1m |  |  |  |  | 3 |
| 6 | 61 | UPS | No | No | 800 | 10 (7/3) | PR | No |  |  |  |  | 23 |
| 7 | 29 | ASPS | No | No | 800 | 29 (19/10) | PR | No |  |  |  |  | 51 |
| 8^*3^ | 44 | exCS | Bone | No | 800 | 23 (18/5) | SD | +, 8m | Subpleural type | 9m (moderate) | Drainage | No | 9 |
| 9 | 49 | UPS | No | No | 400 | 12 (5/7) | PD | No |  |  |  |  | 1 |
| 10 | 59 | UPS | No | No | 200 | 3 (0/3) | PR | No |  |  |  |  | 7 |
| 11 | 73 | LMS | No | No | 400 | 7 (1/6) | PD | No |  |  |  |  | 2 |
| 12 | 67 | AS | Bone, peritoneum | No | 400 | 15 (12/3) | SD | No |  |  |  |  | 1 |
| 13 | 22 | CC | Bone, lymph node | No | 400 | 15 (11/4) | SD | No |  |  |  |  | 1 |
| 14 | 63 | UPS | Kidney | VATS | 400 | 3 (1/2) | SD | No |  |  |  |  | 4 |
| 15 | 68 | UPS | Bone | No | 400 | 8 (2/6) | SD | No |  |  |  |  | 4 |
| 16 | 77 | MFS | Soft tissue | No | 400 | 12 (6/6) | SD | No |  |  |  |  | 3 |
| 17 | 24 | SS | Brain | VATS | 400 | 3 (3/0) | SD | No |  |  |  |  | 1 |
| 18 | 62 | UPS | No | Drainage of pleural effusion | 400 | 5 (3/2) | PD | No |  |  |  |  | 1 |
| 19 | 71 | LMS | Chest wall | No | 400 | 6 (4/2) | SD | No |  |  |  |  | 3 |
| 20 | 69 | exOS | No | No | 400 | 43 (32/11) | PD | No |  |  |  |  | 2 |

AS, angiosarcoma; ASPS, alveolar soft part sarcoma; CC, clear cell sarcoma; exCS, extraskeletal chondrosarcoma; exOS, extraskeletal osteosarcoma; LMS, leiomyosarcoma; m, month(s); PD, progressive disease; PR, partial response; PTX, pneumothorax; PZP, pazopanib; RFA, radio-frequent ablation; SD, stable disease; UPS, undifferentiated pleomorphic sarcoma; VATS, video-associated thoracic surgery; w, week(s).

*1: The patient (no. 2, 73-year-old woman, undifferentiated pleomorphic sarcoma) with bilateral multiple metastases with or without cavitation had been identified before administering pazopanib. For solid metastases, radiofrequency ablation was performed for bilateral lung metastases four times. Then, lobectomy of the left lower lobe was performed. Two months after surgery, pazopanib was administered (800 mg). At that time, there were many metastases with small cavitation (approximately 10 mm) and prominent cavitation in the left lung (S5, 26 mm, central lesion). After the initiation of pazopanib treatment, the patient’s condition was well controlled. However, the prominent cavity increased to 57 mm, though the other cavities did not increase. Subsequently, pneumothorax occurred. This pneumothorax was not related to the previously intervened area. After drainage and pleurodesis, the pneumothorax was maintained for 6 months. However, a second pneumothorax occurred in the same cavity.

*2: Patient no. 4, 15-year-old, male, extraskeletal osteosarcoma: Bilateral multiple metastases without cavitation were identified. Two weeks after the initiation of pazopanib, approximately half of the metastatic lesions degenerated into several cavity. At the same time, the patient complained of chest pain; pneumothorax that occurred from the subpleural lesion of the right lung was detected. Two weeks after drainage, pazopanib was initiated. However, after 2 weeks, an asymptomatic small pneumothorax was noted in the subpleural lesion of the left lung. Subsequently, the continuation of pazopanib was considered difficult. However, repeated pneumothorax occurred on both sides.

*3: Patient no. 8 (44-year-old, male, extraskeletal chondrosarcoma): Under the administration of pazopanib, the lung metastases were controlled as stable disease. Six months after treatment, rapid growth and appearance of new lesions were noted. At the same time, cavitation of the subpleural lesion in the right lung appeared. Owing to the progressive form of the disease, pazopanib was discontinued. One week after cessation, pneumothorax occurred in the right lung from the cavitation area and was treated with drainage. After recovery from pneumothorax, re-initiation of pazopanib was difficult because of the patient’s condition. With an increase in the number of metastatic lesions, several lesions accompanied degenerated cysts. This indicated that these cavitations were not related to the administration of pneumothorax but rather to the natural course of the tumor.
